# Comparative Study of Phytochemistry, Antioxidant and Biological Activities of *Berberis libanotica* Fruit and Leaf Extracts

**DOI:** 10.3390/plants12102001

**Published:** 2023-05-16

**Authors:** Michella Dawra, Nancy Nehme, Marc El Beyrouthy, Alain Abi Rizk, Patricia Taillandier, Jalloul Bouajila, Youssef El Rayess

**Affiliations:** 1 Laboratoire de Génie Chimique, Université de Toulouse, CNRS, INPT, UPS, 3136 Toulouse, France; michelladawra@hotmail.com (M.D.); patricia.taillandier@ensiacet.fr (P.T.); 2 Faculty of Agricultural Engineering and Veterinary Medicine, Lebanese University, Dekwaneh P.O. Box 6573, Lebanon; nancy.nehme@ul.edu.lb; 3 Department of Agriculture and Food Engineering, School of Engineering, Holy Spirit University of Kaslik, Jounieh BP 446, Lebanon; marc.beyrouthy@gmail.com (M.E.B.); alainabirizk@usek.edu.lb (A.A.R.)

**Keywords:** *Berberis libanotica*, phytochemicals, endemic plants, antioxidant activity, antibacterial activity, biological activities

## Abstract

*Berberis libanotica* Ehrenb. ex C.K. Schneider of the Berberidaceae family is an endemic Lebanese plant and is widely used in folk medicine. This study highlights the phytochemical composition and biological activities (in vitro) of fruit and leaf extracts. The two organs were extracted by cold maceration with four solvents of increasing polarity: cyclohexane, dichloromethane, ethyl acetate and methanol. The extracts were screened for their chemical composition by HPLC-DAD to identify and quantify the phenolic compounds. Volatile compounds were detected by GC-MS. The antioxidant capacity through DPPH inhibition was tested. The anti-acetylcholinesterase, antibacterial and anti-proliferative activities were evaluated. Thirteen compounds, including 12 phenolics, were detected in the fruits, whereas 8 phenolic compounds were identified in the leaves. A total of 137 volatile compounds were identified in both organs. At 50 μg/mL, the methanolic leaf extract presented the highest antioxidant capacity, with an inhibition percentage of 54.9%. The dichloromethane fruit extract reduced the acetylcholinesterase activity by 65.3%. The cyclohexane leaf extract reduced the proliferation of the HCT-116 cells by 54.8%, while the dichloromethane fruit extract exhibited the best inhibition against the Caco-2 cells (54%). Interestingly, the minimum inhibitory concentration (MIC) value of the cyclohexane fruit extract against *Salmonella enterica* serovar Kentucky was 2.4 μg/mL, and the MIC value of the cyclohexane leaf extract against *E. coli* was 9.7 μg/mL.

## 1. Introduction

The genus *Berberis* (Berberidaceae) includes about 500 different species and is commonly grown in Europe, North and South America and South Asia. The leaves and fruits can be prepared as food flavorings, juices and teas. *Berberis* species are mainly consumed fresh, dried and used in juice production [1]. These plants are also studied as indicators of habitat degradation in the temperate regions due to their thorny stems and unpalatable shoots [2]. The members of this genus are widely cultivated around the world on account of their high medicinal and ornamental value. A previous study reported the involvement of *B. vulgaris* L. and *B. aetnensis* C. Presl in many domains, such as drugs, foods, beverages and medications [3]. Interestingly, some *Berberis* types have influenced in a good way the neuronal system, suggesting a potential use for the treatment of some neuronal disorders [4]. Chemical investigations proved that the biological activities of *Berberis* species were mainly due to their alkaloid components, such as berberine, oxyacanthine, berbamine and palmatine [5,6]. Particularly, berberine has been intensively studied for its pharmacological properties. The latter was shown to inhibit the migration of melanoma cancer cells, enhance tumor-necrosis-factor-related apoptosis-inducing ligand in breast cancer, and exert a cytotoxic effect against many cell lines [7]. To date, *B. libanotica* Ehrenb. ex C.K. Schneider has been poorly explored from both chemical and biological points of view. It is endemic to Lebanon and Syria and its vernacular name is Lebanon Barberry [8]. It is found in abundance on the slopes of Lebanon and Anti-Lebanon between 1400 and 2000 m and is found in regions such as Arsal, Kfardebian, Jabal Barouk and Ehden. It is an endangered plant due to grazing by the Baladi goats during summer [8]. It has blackish or reddish woody branches, yellow spines, glabrous elliptical lanceolate leaves (10–15 × 3–5 mm) and sessile yellow flowers whose petals and sepals (tepals) resemble each other (4–5 mm). Its fruit is a blackish and oval berry. Blooming time is between May and June [9]. The berry is edible, but it causes digestive disorders in children. *B. libanotica* roots, commonly known as Shalesh El Barbaris, have been used in traditional herbal Lebanese remedies for rheumatic and neuralgic diseases [7,8,9]. Moreover, its leaves are used for the treatment of arthritis and muscular pain [8]. Despite all the hard work carried out on the *Berberis* genus in terms of its anti-inflammation and cardiotonic antiarrhythmic effects, its roles and effects in other biological activities, such as anti-xanthine oxidase and anti-proliferation of cancer cells, have still not been elucidated. The current study was conducted to evaluate the phytochemical profile and biological activities of *B. libanotica* fruit and leaf extracts. A comparison between their corresponding chemical profiles was established in order to better understand the relationship between the identified molecules and the biological activities examined.

## 2. Results and Discussion

### 2.1. Plant Materials and Extraction Yields

As displayed in Table 1, the highest extraction yields were recorded with the methanol (MeOH) extracts of leaves and fruits and corresponded to 16.8% and 15.6%, respectively. The yields of the cyclohexane (CHX) extracts prepared from fruits and leaves were 5.4% and 0.7%, respectively. The poorest yields were registered with the dichloromethane (DCM) extract of leaves (0.2%) and the ethylacetate (EtOAc) extract of fruits (0.1%). No previous studies have assessed the dry extract of *Berberis* species with the EtOAc solvent.

### 2.2. Total Phenolic Content (TPC)

As shown in Table 2, the highest total phenolic content (TPC) was obtained with the methanolic extract of leaves (132.7 mg GAE/g dw), which suggests that the majority of the phenolic compounds or the most concentrated ones were polar. The MeOH extract of fruits also recorded the highest score (94.8 mg GAE/g dw). The EtOAc extract showed the second most important phenolic content for both leaves and fruits with 122.0 and 68.9 mg GAE/g dw, respectively, followed by the DCM extracts of leaves and fruits with 65.8 and 17.3 mg GAE/g dw, respectively. The CHX extracted the lowest amount of phenolic compounds from both organs, with 32.8 and 16.9 mg GAE/g dw for leaves and fruits, respectively. The latter contained the non-polar phenolic compounds. It is important to highlight that for both fruits and leaves, the TPC obtained in the MeOH extracts was higher than those obtained from the extracts of other *Berberis* organs. El Hosry et al. (2016) [10] demonstrated that the TPC of the methanolic extract (80% MeOH-20% H_2_O) of *B. libanotica* roots was 50 mg GAE/g dw, which was 2.65 and 1.9 times lower than the values obtained with the leaves and fruits in the current study. However, the TPC of their MeOH-fruits extract (187 mg GAE/g dw) was two times greater than the amount obtained in the current study (94.8 mg GAE/g dw). The differences can be attributed to many factors, such as the plant development stage, ecological conditions and geographic coordinates. 

### 2.3. Chromatographic Fingerprint Analyses by High-Performance Liquid Chromatography Coupled with Diode Array Detector (HPLC-DAD)

**Figure 1:**
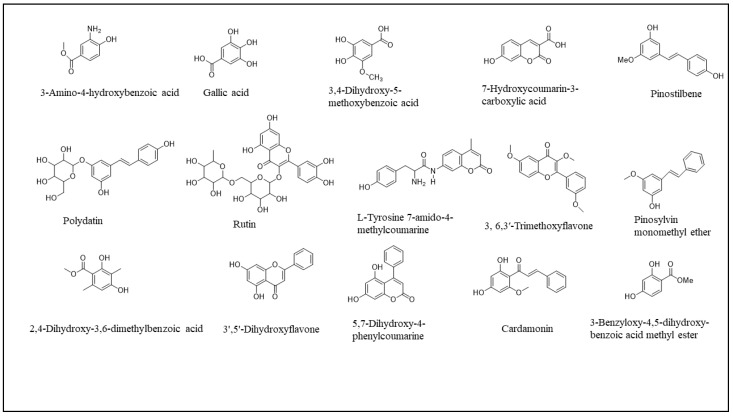
Chemical structures of the compounds detected in the extracts of *B. libanotica* leaves and fruits by HPLC-DAD analysis at 280 nm.

**Figure 2:**
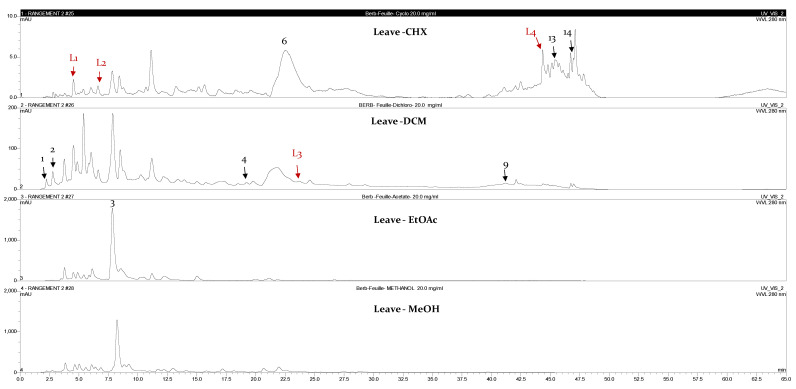
HPLC-DAD chromatograms at 280 nm of the CHX, DCM, EtOAc and MeOH extracts of *B. libanotica* leaves: 3-amino-4-hydroxybenzoic acid (**1**); gallic acid (**2**); 3,4-dihydroxy-5-methoxybenzoic acid (**3**); L-tyrosine 7-amido-4-methylcoumarine (**4**); rutin (**6**); 3′,5′-dihydroxyflavone (**9**); 3-benzyloxy-4,5-dihydroxy-benzoic acid methyl ester (**13**); and pinosylvin monomethyl ether (**14**). The red arrows designate the suggested chemical classes in Table 4.

**Figure 3:**
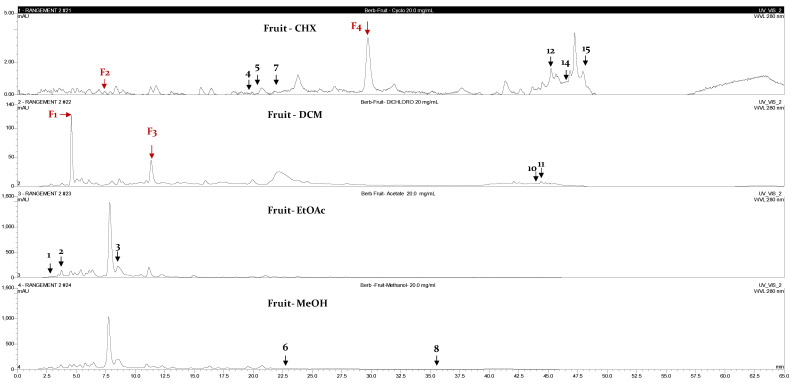
HPLC-DAD chromatograms at 280 nm of the CHX, DCM, EtOAc and MeOH extracts of *B. libanotica* fruits: 3-amino-4-hydroxybenzoic acid (**1**); gallic acid (**2**); 3,4-dihydroxy-5-methoxybenzoic acid (**3**); L-tyrosine 7-amido-4-methylcoumarine (**4**); 7-hydroxycoumarin-3-carboxylic acid (**5**); rutin (**6**), polydatin (**7**), 2,4-dihydroxy-3,6-dimethylbenzoic acid (**8**); 5,7-dihydroxy-4-phenylcoumarine (**10**); cardamonin (**11**); pinostilbene (**12**); pinosylvin monomethyl ether (**14**); and 3,6,3′-trimethoxyflavone (**15**). The red arrows designate the suggested components.

The identification of the compounds by HPLC-DAD was based on the comparison of the HPLC retention times and the DAD spectra to those obtained with the co-injection of some commercial authentic standards. Thirteen compounds, including 12 phenolics and one methoxyphenol, were detected in the fruits, whereas 8 phenolic compounds were detected in the leaves (Figure 1). The amount of each compound was expressed in milligrams per gram of the corresponding total extract (Table 3). Interestingly, 90% of the detected compounds were found for the first time in *B. libanotica* leaves and fruits. Gallic acid (**2**), which was previously detected in the fruits by El Hosry et al. [10], was found in the EtOAc extract of both leaves and fruits at the same amount, 0.2 mg/g. As shown in Figure 2 and Figure 3, the eight chromatograms demonstrated that more than half of the detected compounds (57.6%) were extracted by the non-polar solvents. The 3-amino-4-hydroxybenzoic acid (**1**) was mainly extracted from leaves and fruits by the MeOH solvent at 1.3 and 1.2 mg/g, respectively. The 3,4-dihydroxy-5-methoxybenzoic acid (**3**) was detected in the EtOAc extracts of both organs (239.9 and 164.4 mg/g in the leaves and fruits, respectively). L-Tyrosine 7-amido-4-methylcoumarine (**4**) was detected in the DCM extract of leaves at a concentration of 4.8 mg/g and in the CHX extract of fruits at 0.2 mg/g. Some compounds were only present in the fruits, such as 7-hydroxycoumarin-3-carboxylic acid (**5**); polydatin (**7**); 2,4-dihydroxy-3,6-dimethylbenzoic acid (**8**); 5,7-dihydroxy-4-phenylcoumarine (**10**); cardamonin (**11**); pinostilbene (**12**); and 3,6,3′-trimethoxyflavone (**15**). Two compounds were exclusively present in the leaves: 3’,5’-dihydroxyflavone (**9**) and 3-benzyloxy-4,5-dihydroxy-benzoic acid methyl ester (**13**). Interestingly, rutin (**6**) was found in the CHX and MeOH extracts of the leaves, but at a higher concentration in the CHX (4.0 mg/g > 2.2 mg/g). Cardamonin (**11**) was only present (5.5 mg/g) in the DCM extract of fruits. Pinosylvin monomethyl ether (**14**) appeared only in the CHX extracts of both leaves and fruits, but was more concentrated in the leaves (1.1 mg/g > 0.7 mg/g). As displayed in Table 4, some compounds present at non-negligible amounts in the extracts were not identified because their commercial authentic standards were not available; hence, the chemical class to which they belong was suggested based on their maximum absorption and on some literature information of *B. libanotica* and related species.

### 2.4. Gas Chromatography Mass Spectrometry (GC-MS) Analysis

Thirty-one compounds were detected by GC-MS before derivatization and one hundred and six additional ones after derivatization (Table 5). This is the first study that analyzes the volatile compounds of the organic extracts of *B. libanotica*. EtOAc extracts of both organs did not contain any volatile compounds. In total, 47.5% of the detected compounds were present in the CHX extract, underlining their nonpolar nature. A total of 92 compounds were exclusively detected in the leaves, whereas 23 were only detected in the fruits and 22 compounds existed in both organs. Some fatty acids, such as oleic acid (13), myristic acid (46′), pentadecanoic acid (53′), α-linolenic acid (57′), stearic acid (68′), arachidic acid (70′), behenic acid (77′) and lignoceric acid (89′), and some sterols, such as β-sitosterol (26), campesterol (101′) and α-amyrin (103′), were also found in the fruits of the Russian *B. vulgaris* [13]. Based on Table 5, some compounds existed in more than one extract, such as palmitic acid (9) and 1-monopalmitine (75′). This behavior is attributed to the extraction process intended to soften and break the plant’s cell walls in order to release the soluble phytochemicals. The amount of each compound will then depend on its affinity to each extract, mainly its polarity and solubility. 

### 2.5. Antioxidant Capacity (DPPH Assay)

**Figure 4:**
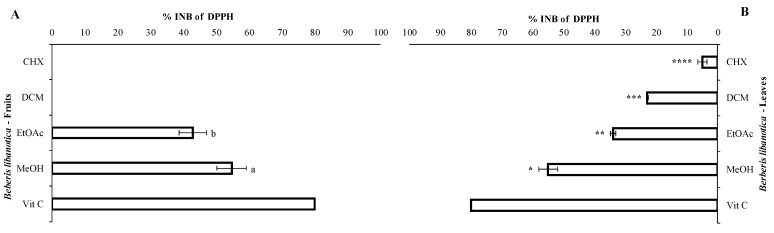
Antioxidant assay of the extracts of *B. libanotica* aerial part (fruits (**A**) and leaves (**B**)) at 50 μg/mL and the standard Vit C at 5.9 μg/mL. The results are expressed as the inhibition percentages (% INB) and are the means of quadruplicate experiments (±SD). a,b,*,**,***,****: different letters and symbols mean significant differences between the values obtained with the extracts of the same organ (fruits and leaves, respectively) according to Tukey’s test (*p* ≤ 0.05).

The strongest DPPH inhibition was obtained with the MeOH extracts of both leaves and fruits (54.9 and 54.7%, respectively (Figure 4)). EtOAc extracts showed an inhibition percentage of 33.8% with the leaves and 42.8% with the fruits (*p* ≤ 0.05). Concerning the non-polar solvents, no inhibition was registered with the CHX and DCM extracts of the fruits; however, those prepared from the leaves showed a weak inhibition of 22.8% and 4.9% with the DCM and CHX extracts, respectively (*p* ≤ 0.05). In the non-polar extracts of the leaves, it can be suggested that 3′,5′-dihydroxyflavone (9) and 3-benzyloxy-4,5-dihydroxybenzoic acid methyl ester (13) of Table 3, in addition to thymol (10′), carvacrol (12′), 2-methyl-6-tert-butylphenol (19′) and vanillic acid (37′)of Table 5, were responsible, along with other molecules, for DPPH inhibition. Koldaş et al. showed that 3,4-dihydroxybenzaldehyde (29′), tert-butylhydroquinone (25′) and caffeic acid (65′) exhibited an important inhibition of DPPH activity (66.9%) at 50 μg/mL. The latter were detected in the DCM extract of the leaves (Table 5) [14]. Asha et al. showed that lupeol (31, Table 5) exhibited a considerable dose-dependent inhibition of DPPH activity with an IC_50_ of 30 mg/mL and was slightly less active than the standard quercetin (IC_50_ = 21 mg/mL) [15]. The MeOH extracts which exhibited the highest antioxidant activity contained 3-amino-4-hydroxybenzoic acid, 3,4-dihydroxy-5-methoxybenzoic acid, rutin and 2,4-dihydroxy-3,6-dimethylbenzoic acid (Table 3). The good quenching activity of the MeOH extracts resulted from the presence of these phenolics along with other molecules. It is important to highlight that the pharmacological potential of many plant materials is presumably due to the capability of polyphenols to interact with important cellular processes in which key enzymes, such as cyclooxygenase, lipooxygenase, phospholipase A2, NADH-oxidase or glutathione reductase, are involved. Bonesi et al. demonstrated that *B. libanotica*-MeOH root extract had an IC_50_ value of 59 μg/mL [3]. Therefore, the MeOH extracts of both the leaves and fruits of the current study showed a better antioxidant activity with an IC_50_ of 45.6 μg/mL.

### 2.6. Anti-Acetylcholinesterase Activity

**Figure 5:**
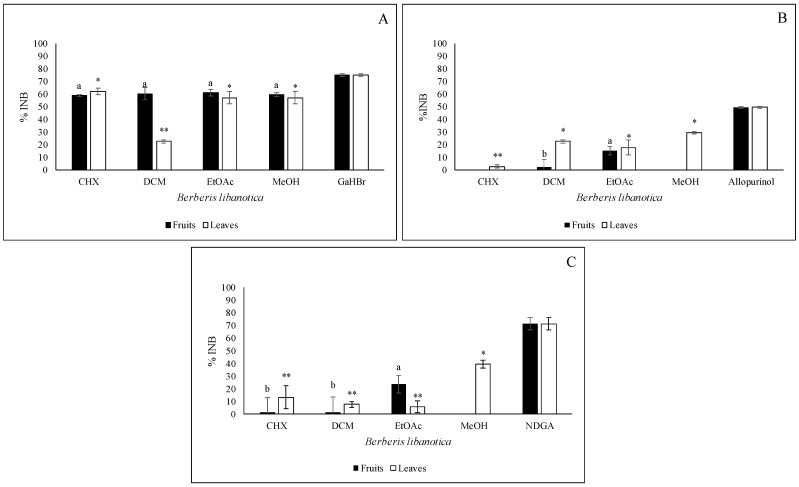
Anti-AChE (**A**), anti-XOD (**B**) and anti-inflammatory or anti-15 LOX (**C**) activities of the *B. libanotica* extracts (leaves and fruits) tested at 50 μg/mL. The results are expressed as the inhibition percentages (% INB) and are the means of quadruplicate experiments (±SD). a,b,*,**: different letters and symbols mean significant differences between the values obtained with the extracts of the same organ (fruits and leaves, respectively) according to Tukey’s test (*p* ≤ 0.05).

The results of the anti-acetylcholinesterase activity (anti-AChE) are illustrated in Figure 5A. A similar inhibition percentage was registered for the MeOH (leaves: 57.3%; fruits: 59.7%), EtOAc (leaves: 57.3%; fruits: 61.3%) and CHX (leaves: 62.4%; fruits: 59%) extracts. The DCM extracts showed different behaviors. The DCM fruit extract presented an inhibition of 60.5%, while the DCM leaf extract had an inhibition of only 22.9%. Bonesi et al. (2013) [3] isolated and tested pure alkaloids that were responsible for significant AChE inhibition, such as berberine and palmatine [3].

### 2.7. Anti-XOD Activity

The MeOH extract of the leaves exhibited the highest anti-xanthine oxidase activity (anti-XOD), with an inhibition percentage of 29.6% (Figure 5B), followed by the DCM and the EtOAc extracts (22.7% and 18%, respectively). A poor inhibition was recorded by the CHX extract (2.9%). No activity was detected with the CHX and MeOH extracts prepared from the fruits. The EtOAc and DCM extracts of the fruits inhibited the enzyme by 15.5 and 2.6%, respectively (*p ≤* 0.05). It is important to highlight that no previous studies have evaluated the anti-XOD activity of these extracts.

### 2.8. Anti-Inflammatory Activity

As shown in Figure 5C, the best anti-inflammatory activity was obtained with the methanolic leaf extracts (39.6%), followed by the CHX (13.1%), the DCM (7.8%) and the EtOAc (5.7%) leaf extracts. Concerning the fruits, the highest inhibition was recorded with the EtOAc extract (23.7%), followed by the DCM (1.7%) and the CHX extract (1.2%). No inhibition was shown with the MeOH extract. No previous studies have investigated the anti-inflammatory activity of the *B. libanotica* extracts against the 15-LOX activity. However, Zhang et al. (2019a) [16] showed that chlorogenic acid (98′), ferulic acid (62′) and caffeic acid (65′) had strong inhibitory potential against soybean lipoxygenase with IC_50_ values of 61.3, 410.2 and 95.6 μmol/l. Esculetin (63′) is considered to be a good analgesic. It significantly inhibited the 5-LOX enzyme at a dose of 20 mg/Kg and reduced the inflammation and pain in rats [17].

### 2.9. Anti-Proliferation Activity

**Figure 6:**
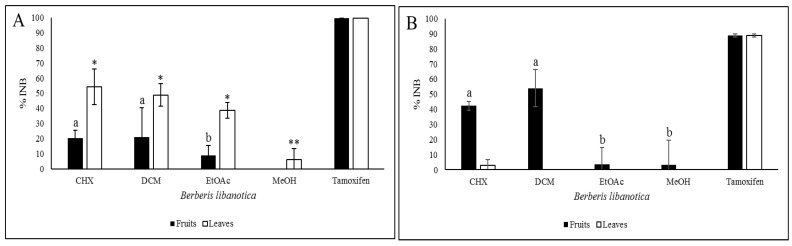
Growth inhibition of the HCT-116 (**A**) and Caco-2 cells (**B**) by the *B. libanotica* extracts tested at 50 μg/mL. Tamoxifen was used as a standard at 37.1 μg/mL. The results are expressed as the inhibition percentages (% INB) and are the means of quadruplicate experiments (±SD). Different letters and symbols mean significant differences between the values obtained with the extracts of the same organ according to Tukey’s test (*p* ≤ 0.05). The letters are attributed to the fruits and the symbols to the leaves.

The anti-proliferation activity of the *B. libanotica* extracts prepared at 50 μg/mL was tested on two lines of cancer cells: Caco-2 and HCT-116. Tamoxifen was used as a positive control. The CHX extract of leaves inhibited the proliferation of the HCT-116 cells by 54.2% (Figure 6A) and the Caco-2 cells by 3% (Figure 6B). The DCM leaf extracts inhibited the proliferation of the HCT-116 cells by 48.8%. This was followed by the EtOAc (38.7%) and the MeOH (6.2%) ones (Figure 6A). Conversely, no inhibition was registered for the Caco-2 cells with the DCM, EtOAc and MeOH leaf extracts. Concerning the fruits, the highest growth inhibition of the HCT-116 cells was registered with the DCM extract (21%). It was followed by the CHX (20%) and the EtOAc (8.8%) ones. No inhibition was recorded with the MeOH extract. The highest inhibition for the Caco-2 cells was obtained with the DCM extract (54%), followed by the CHX (42.3%), then the two polar extracts with 3.5 and 3.2% for the EtOAc and the MeOH extracts, respectively (Figure 6B). The proliferation inhibition of the HCT-116 cells can be partially explained by the presence of *β*-sitosterol (26, Table 5), which was previously shown to reduce the growth of the HCT-116 cells by 60% at 20.7 μg/mL [18]. β-amyrin (29, Table 5) and ursolic acid (106′, Table 5) inhibited the HCT-116 cells and gave IC_50_ values of 8.9 μg/mL [19] and 16.9 μg/mL [20], respectively.

### 2.10. Antibacterial Activity

The EtOAc and MeOH leaf extracts did not exhibit any antibacterial activity. The CHX extract showed an important antibacterial activity against *E. coli* ATCC 8739, *Salmonella enterica* serovar Enteritidis and *L. monocytogenes* ATCC 19115 with low MIC values of 9.7, 19.5 and 19.5 μg/mL, respectively (Table 6). The DCM extract showed good inhibition against the three serovars of *Salmonella* (Enteritidis, Kentucky and Infantis) and against *L. monocytogenes* ATCC 19115 with a MIC value of 19.5 μg/mL for *Salmonella* Infantis and 39 μg/mL for the others. The fruit extracts showed a different antibacterial activity against the same studied strains. The two polar extracts exhibited the lowest antibacterial activity and the best one was obtained with the EtOAc extract against *S. aureus* ATCC 25923 with a MIC value of 78.1 μg/mL. The DCM extract inhibited the growth of *Salmonella* Enteritidis with a MIC value of 39 μg/mL. The CHX extract significantly inhibited *Salmonella* Kentucky and Enteritidis (MIC values of 2.4 and 9.7 μg/mL, respectively) as well as both strains of *L. monocytogenes* (MIC values of 2.4 and 4.8 μg/mL for ATCC 19115 and fish filet, respectively) (Table 7).

The antibacterial activity of these extracts can be explained by the presence of some compounds known for their antimicrobial activity such as phytol (10, Table 5), which is present in the CHX and DCM extracts of leaves and fruits. It is known for its antibacterial activity against *L. monocytogenes* strains [21]. The antibacterial activity of the CHX and DCM extracts was relevant against some strains more than others. This might be attributed to several resistance mechanisms that make some bacterial strains less sensitive to the same molecules or extracts [22]. The fumaric acid and caffeic acid (16′and 65′ of Table 5) present in the DCM leaf extract were previously shown to exert antimicrobial properties in vitro against several bacterial and fungal strains [23]. The cajaninstilbene acid and chlorogenic acid (95′and 98′ of Table 5), also present in the DCM leaf extract, were previously shown to exert a significant antibacterial activity against some *S. aureus, E. coli* and *Salmonella* strains with MIC values ranging between 16 and 80 μg/ml [24,25]. The antibacterial activity of the CHX and DCM extracts can also be attributed to some phenolic compounds, such as pinosylvin monomethyl ether, present in these extracts (Table 3).

### 2.11. Statistical Analysis

**Figure 7:**
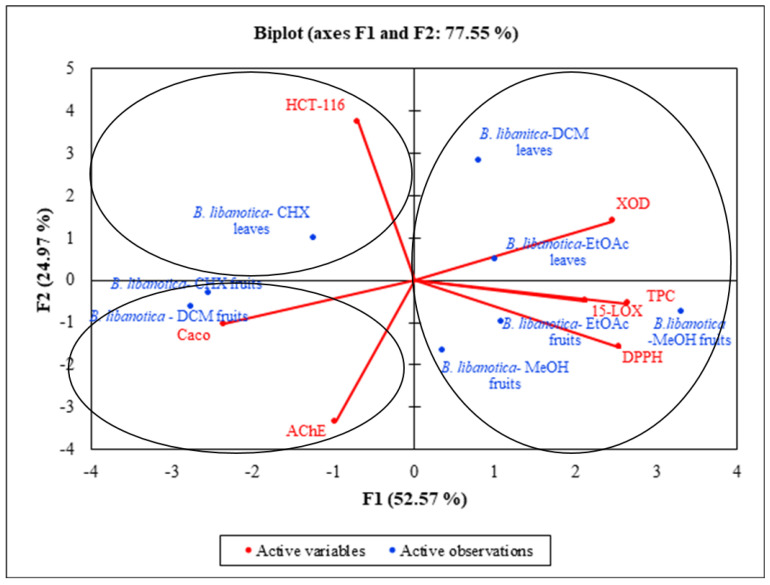
Contribution of the variable factors to the principal component analysis (%).

The principal component analysis (PCA) was performed to find the relationship between the different biological activities and the phytochemical composition of the extracts. Table 8 shows that the axes of inertia have been hidden from this analysis. The percentage of total variation was recorded at 77.5% and proven by the structuring accessions in Figure 7. The axes were retained because they expressed 52.5% (PC_1_) and 24.9% (PC_2_). Simultaneously, the loadings in the PCA loading plots expressed how good the correlation was between the major components and the original variables studied. There was a very good correlation between the antioxidant activity and the TPC. PC_1_ was highly correlated with the TPC with loading of 0.91 (Table 9). As previously shown in Section 2.2, the polar extracts of both organs presented the highest polyphenol content and gave the best DPPH inhibition percentages. Moreover, a good correlation was established between the anti-XOD activity, the anti-15-LOX activity and the TPC. PC_1_ was highly correlated with the TPC with loadings of 0.8 and 0.7, respectively (Table 9). The second axis was well correlated with HCT-116 with a loading of 0.8 (Table 8). When applying the principal component analysis, it seemed that there was a discriminate structure. The oval forms grouped the different extracts in three classes: C1 (*B. libanotica* leaves—DCM; *B. libanotica* leaves—EtOAc; *B. libanotica* leaves—MeOH; *B. libanotica* fruits—EtOAc; and *B. libanotica* fruits—MeOH), C2 (*B. libanotica* leaves—CHX) and C3 (*B*. *libanotica* fruits—CHX and *B. libanotica* fruits—DCM). Since the two plots (biplot) were gathered together, it can be noticed that the high TPC and antioxidant, and anti-XOD and anti-15-LOX activities were related to many extracts in the following way: *B. libanotica* leaves—DCM; *B. libanotica* leaves—EtOAc; *B. libanotica* leaves—MeOH; *B. libanotica* fruits—EtOAc; and *B. libanotica* fruits—MeOH extracts. In addition, the *B. libanotica* leaves—CHX; *B. libanotica* fruits—CHX; and *B. libanotica* fruits—DCM (poor in TPC); and the anti-acetycholinesterase activity (AChE) as well as the anti-proliferation activity (HCT-116 and Caco-2 cells) were located symmetrically in the negative side of the PC_1_ axis, which suggests that the highest anti-proliferation and anti-acetylcholinesterase activities of these extracts were not only related to their phenolic compounds.

## 3. Materials and Methods

### 3.1. Chemicals and Plant Materials

All the chemicals used in this study were purchased from Sigma-Aldrich (Saint-Quentin, France). The analytical standards used for the assignation of the main phenolic compounds found in the plant extracts were 3-amino-4-hydroxybenzoic acid; gallic acid; 3,4-dihydroxy-5-methoxybenzoic acid; L-tyrosine 7-amido-4-methylcoumarine; rutin; polydatin; 2,4-dihydroxy-3,6-dimethylbenzoic acid; 3′,5′-dihydroxyflavone; 5,7-dihydroxy-4-phenylcoumarine; cardamonin; pinostilbene; 3-benzyloxy-4,5-dihydroxy-benzoic acid methyl ester; pinosylvin monomethyl ether; and 3, 6,3′-trimethoxyflavone. Samples of the aerial part of *B. libanotica* were obtained from Kfardebian (elevation 1220 m) in October 2018, and authenticated by Dr. Marc El Beyrouthy (expert in botany and owner of the company *Nature by Marc Beyrouthy*). A specimen was deposited in the Herbarium of Botany, School of Engineering of the Holy Spirit University of Kaslik, Lebanon under the registry number MNIIIb180c.

### 3.2. Preparation of the Extracts

After two weeks of drying in shade at ambient temperature, the dried leaves and fruits of *B. libanotica* were ground into powder with a particle size of 0.8 mm [26]. A cold maceration was performed using four solvents with increasing polarity (CHX, DCM, EtOAc and MeOH) to yield the four organic extracts. Over 2 h, 100 g of powder was successively extracted with 2 l of each solvent at an agitation of 300 rpm without heating. The filtrates were recovered after filtration through whatman filter papers (Fisher, France). The extracts were obtained by evaporating the solvent under vacuum at 35 °C and then were stored at ambient temperature until further analysis.

### 3.3. Total Phenolic Content (TPC)

The total phenolic content (TPC) of each extract was evaluated using the Folin–Ciocalteu (F.C) method at 765 nm as previously mentioned by Dawra et al. [26]. The calibration curve was determined using the standard “gallic acid” at a concentration ranging between 0 and 115 µg/mL. Results were displayed as mg of gallic acid equivalents (GAE)/g dw.

### 3.4. Chromatographic Fingerprint Analyses by High-Performance Liquid Chromatography Coupled with Diode Array Detector (HPLC-DAD)

The HPLC analysis was carried out using an ultimate 3000 pump-Dionex and Thermos Separation product detector DAD model (Thermo Fisher Scientific, USA). The separation was carried out on an RPC18 reversed-phase column (Phenomenex, Le Pecq, France), 25 cm × 4.6 mm and particle size of 5 μm, thermostated at 25 °C, as previously reported by Dawra et al. [26]. The elution was performed at a flow rate of 1.2 mL/min, using a mobile phase consisting of MilliQ water (pH 2.6) (solvent A) and acidified water/MeCN (20:80 *v*/*v*) (solvent B). The pH was adjusted to 2.6 using glacial acetic acid (ACS, 99.7%, Thermo Scientific Chemicals). The samples were eluted by the following linear gradient: from 12% B to 30% B for 35 min, from 30% B to 50% B for 5 min, from 50% B to 88% B for 5 min and finally from 88% B to 12% B for 15 min. The samples were prepared at a concentration of 20 mg/mL using the acidified water/MeCN (80:20 *v*/*v*) mixture, and then filtered through a Millex-HA 0.45 µm syringe filter (Sigma Aldrich, Saint-Quentin-Fallavier, France). An amount of 20 μL of each sample was injected and the detection was registered at 280 nm. The identification of the compounds by HPLC-DAD was based on the comparison of the retention times and the DAD spectra at the maximum absorbance of each compound to those obtained with the co-injection of some commercial authentic standards. Quantification was performed using the corresponding calibration curves at the maximum UV absorbance of each compound as indicated in Table 3.

### 3.5. Gas Chromatography Mass Spectrometry (GC-MS) Analysis

The identification of the volatile compounds of the organic extracts, before and after derivatization, was conducted using the protocol described by Dawra et al. [26]. The analyses were performed using an Agilent gas chromatograph 6890 coupled to a 5975 mass detector. The 7683 B auto sampler injected 1 μL of each extract. A fused silica capillary column DB-5 MS (30 m × 0.25 mm internal diameter, film thickness 0.25 μm) (Supelco, Sigma-Aldrich, Darmastadt, Germany) was used. The column temperature was initially 35 °C before being gradually increased to 85 °C at 15 °C/min, held for 20 min isothermally at 85 °C, raised to 300 °C at 10 °C/min and finally held for 5 min at 300 °C. Helium (purity 99.99%) was used as a carrier gas at a flow rate of 0.8 mL/min. Mass spectra were registered at 70 eV with an ion source temperature held at 310 °C and a transfer line heated at 320 °C. The mass scanning was performed from 50 to 1200 amu. The main goal was to find the maximum similarity in terms of spectra between the compounds found in the extracts and those suggested by the NIST database. The identification of the components was carried out by using the mass spectra in comparison with those obtained in NIST08 (National Institute of Standards and Technology, https://www.nist.gov/ accessed on 21 September 2021), using AMDIS software, and the retention time was used to facilitate many tasks. For analysis purposes, each sample was dissolved with its own solvent (5 mg/mL) before injection. The derivatization method consisted of dissolving 5 mg of each extract in 1 mL of its own solvent except for the MeOH extract. The latter was dissolved in MeCN. After that, 150 μL of BSTFA and 1.5 μL of TMSC were added to the solution. The mixture was agitated for 30 s in order to increase the solubility. The reaction mixture was maintained at 40 °C for 30 min. An amount of 10 μL of each derivatized solution was injected into the GC-MS and analyzed as previously reported.

### 3.6. Antioxidant Capacity (DPPH Assay)

The antioxidant scavenging capacity was assessed using the DPPH assay as reported by Dawra et al. [26]. An amount of 180 μL of 0.2 mM methanolic DPPH solution was added to 20 μL of the diluted plant extract (500 μg/mL) in a 96-well microplate (Micro Well, Thermo Fisher Scientific, Asian, France). The final concentration of the extract in each well was 50 μg/mL. After an incubation period of 25 min at room temperature, the absorbance of each sample (A_sample_) was measured at 515 nm. The A_blank_ was measured without the extract. Vitamin C was used as a positive control. The inhibition percentage of DPPH was obtained using the following equation: $\;\%\;\mathrm{INB}=100\times \left(\frac{Ablank-Asample}{Ablank}\right)$.

### 3.7. Anti-Acetylcholinesterase Activity

The anti-acetylcholinesterase (AChE) activity was tested using Ellman’s procedure as previously reported by Dawra et al. [26]. For this, 50 μL of 0.1 mM sodium phosphate buffer (pH = 7.5), 125 μL of 5,5′-dithio-bis-(2-nitrobenzoic acid) (DTNB), 25 μL of diluted plant extract (500 μg/mL) and 25 μL of enzyme solution (493. 2 U) were mixed and incubated for 15 min at 25 °C in a 96-well microplate. Then, 25 μL of acetylthiocholine (ACTHI) was added. The final blend was incubated for 25 min at 25 °C and the absorbance was measured at 421 nm. The A_blank_ was measured without the extract. Galanthamine dibromide (GaHBr) was used as a positive standard. The inhibition percentage of the AChE activity was determined as follows: $\;\%\;\mathrm{INB}=100\times \left(\frac{Ablank-Asample}{Ablank}\right)$.

### 3.8. Anti-XOD Activity

The XOD activity using xanthine as the substrate was tested by following the procedure previously described by Dawra et al. [27]. The xanthine solution was prepared at a concentration of 1 mM in 25 mL of 0.1 mM sodium phosphate buffer (pH = 7.5). The concentration of the XOD enzymatic solution was 0.1 U/mL and was obtained by diluting the XOD from cow’s milk. Then, 50 μL of diluted plant extract (200 μg/mL) was added to 60 μL of 70 mM sodium phosphate buffer (pH = 7.5) and 30 μL of the enzymatic solution. The assay mixtures were prepared in a 96-well quartz microplate. The latter was incubated at 25 °C for 15 min. Then, 60 μL of the substrate solution were added and the absorbance was measured at 295 nm after 5 min. The final concentration of the extract in each well was 50 μg/mL. Allopurinol was used as a positive control. The inhibition percentage of the XOD activity was calculated as follows: $\;\%\;\mathrm{INB}=100\times \left(\frac{Ablank-Asample}{Ablank}\right)$.

### 3.9. Anti-Proliferation Activity

The cytotoxicity of the plant extracts was estimated on two different types of human colon cancer cells (HCT-116 and Caco-2). The test was performed as reported by Dawra et al. [26]. The cell growth was assessed by the MTT assay. MTT is a yellow water-soluble tetrazolium salt that is reduced by the mitochondrial dehydrogenases of intact cells to a purple formazan product. The cells were introduced in a 96-well plate at 3.10^4^ cells/well in a volume of 100 μL of a suitable culture medium. After that, 100 μL of the same culture medium containing the plant extract was added. The final concentration of the extract in each well was 50 μg/mL. The culture media used were, respectively, RPMI 1640 for the HCT-116 cells and Dulbecoo’s modified Eagle’s medium GlutaMAX (DMEM) for the Caco-2 cells (Sigma Aldrich, Saint Louis, Missouri, USA). Tamoxifen was used as a positive reference. The microplate was incubated at 37 °C for 48 h. The supernatant was then removed and 50 µL of the MTT solution was added, followed by an incubation of 40 min. The absorbance was measured at 605 nm. The inhibition percentage of the cells’ proliferation was calculated as follows: $\;\%\;\mathrm{INB}=100\times \left(\frac{Ablank-Asample}{Ablank}\right)$.

### 3.10. Anti-Inflammatory Activity

This activity was carried out using the method described by Dawra et al. [27]. The test was performed in a 96-well quartz microplate. For this, 150 μL of 100 mM phosphate buffer (pH 7.4) was added to each well with 60 μL of linoleic acid (3.5 mmol/L), 20 μL of 15-LOX (Soybean 500 U) and 20 μL of the extract solution at a concentration of 250 μg/mL. The mixture was incubated at 25 °C for 10 min and the absorbance was determined at 234 nm. Nordihydroguaiaretic acid (NDGA) was used as a positive control. The final concentration of the extract in each well was 50 μg/mL. The enzyme activity inhibition percentage was calculated as follows: $\;\%\;\mathrm{INB}=100\;\times \;\left(\frac{Ablank-Asample}{Ablank}\right)$.

### 3.11. Antimicrobial Activity

The Gram-positive strains used in this work were *Staphylococcus aureus* ATCC 25923, *Listeria monocytogenes* ATCC 19115 and *Listeria monocytogenes* isolated from “fish-filet” at the Lebanese Agricultural Research Institute (LARI)-Lebanon. The Gram-negative ones were *Escherichia coli* ATCC 8739 and the Kentucky, Infantis and Enteritidis serotypes of *Salmonella enterica* isolated at LARI from chicken samples collected from slaughterhouses. A bacterial suspension of 2.10^8^ CFU/mL (0.5 McFarland standard) was prepared for each strain in a Mueller Hinton Broth (MHB) [28]. A serial microdilution in a 96-well microplate was carried out in order to determine the minimum inhibitory concentration (MIC) values of the *B. libanotica* extracts. Each well first contained 100 μL of MHB. The dried extracts were dissolved in pure DMSO to a concentration of 5 mg/mL. Then, the extract solutions were half-diluted with MHB to obtain a concentration of 2.5 mg/mL. Subsequently, 100 μL of the latter was placed in the first well and a serial dilution was conducted in order to reach the following concentrations in each row: 1250, 625, 312.5, 156.2, 78.1, 39, 19.5, 9.7, 4.8, 2.4 and 1.2 μg/mL. After that, 100 μL of each bacterial strain tested was added to the extract solutions, leading to an initial bacterial concentration of 10^8^ CFU/mL in each well. A mixture of 100 μL of DMSO and 100 μL of the bacterial strain tested was used as the negative control, while the positive one contained 100 μL of MHB and 100 μL of the bacterial strain. The optical density was determined at time 0 min, and again after an overnight incubation at 37 °C (24 h) using a Multiskan Sky Microplate Spectrophotometer (Thermo Fisher Scientific, Waltham, MA, USA).

### 3.12. Statistical Analysis

All measurements were carried out in quadruplicate. The results were subjected to a multi-way analysis of variance, and the mean comparisons were performed by a Tukey’s multiple range test using SPSS version 20.0 (Statistical Package for the Social Sciences, Inc., Chicago, IL, USA). The differences between means were considered significant at *p*-value < 0.05. The linear correlation coefficient (R^2^) was determined to establish the relationship between the TPC and the antioxidant or any other biological activity. The PCA was conducted using XLSTAT (version 2020.1, Addinsoft, Pearson edition, Waltman, MA, USA) for a better discrimination between the studied parameters.

## 4. Conclusions

In conlusion, the present work elucidated the chemical composition and the biological activities of *B. libanotica* Ehrenb. ex. C.K. Schneider grown in Lebanon. The HPLC-DAD enabled the assignation of 13 aromatic compounds in the fruits and 8 in the leaves. The volatile compounds were determined by GC-MS analysis. In total, 45 compounds were detected in the fruits and 114 in the leaves. Based on the conducted experiments at 50 μg/mL and the PCA results, the MeOH extract of the *B. libanotica* leaves presented the highest capacity to inhibit the DPPH (54.9%), which was correlated to its polyphenol content (137.2 mg GAE/g dw). It also registered a moderate inhibition of the XOD and 15-LOX enzymes and gave inhibition percentages of 39.6 and 29.6%, respectively. The DCM extract of the *B. libanotica* fruits showed good anti-acetylcholinesterase and anti-Caco-2 proliferation activities, with inhibition percentages of 65.3 and 54%, respectively. The CHX extract of the *B. libanotica* leaves registered a good cytotoxic activity against the HCT-116 cells, causing a growth inhibition of 54.8%. The CHX extracts of both organs exhibited an interesting antibacterial activity with very low MIC values against Gram-positive and Gram-negative bacteria, particularly *Salmonella* Enteritidis, *Salmonella* Kentucky, *E. coli* and *L. monocytogenes*. The findings obtained encourage us to search for the bioactive compounds that are responsible for the biological activities, and more particularly the antibacterial one that was noticeable, using silica gel fractionation followed by a preparative reversed-phase chromatography.

## Figures and Tables

**Table 1:** Extraction yields of the aerial part of *B. libanotica* Ehrenb. ex C.K. Schneider (leaves and fruits).

Extraction Yields (dw %)		
	Leaves	Fruits
CHX	0.7	5.4
DCM	0.2	0.8
EtOAc	0.5	0.1
MeOH	16.8	15.6

**Table 2:** The total phenolic content of the extracts of the aerial part of *B. libanotica* (mg GAE/g of dw).

TPC (mg GAE/g of dw)		
	Leaves	Fruits
CHX	32.8 ± 1.6 ^c^	16.9 ± 4.3 ^+++^
DCM	65.8 ± 1.8 ^b^	17.3 ± 0.7 ^+++^
EtOAc	122.0 ± 1.5 ^a^	68.9 ± 2.5 ^++^
MeOH	132.7 ± 1.9 ^a^	94.8 ± 3.3 ^+^

Note: ^a,b,c,+,++,+++^ the different superscripts means significant differences between the values obtained with the extracts of the same organ according to Tukey’s test (*p* ≤ 0.05). The letters and symbols refer to the leaf and fruit organs, respectively. Means values ± SD (*n* = 4).

**Table 3:** Quantification of the compounds detected in the extracts of *B. libanotica* by HPLC-DAD analysis at 280 nm.

						Concentration (mg/g of Extract)							
						Leaves				Fruits			
№	tR (min)	λ_max(nm)_	Type of phenolic compound	Compound	Calibration curves	CHX	DCM	EtOAc	MeOH	CHX	DCM	EtOAc	MeOH
1	2.2	281	Derivative of the p-hydroxybenzoic acid	3-Amino-4-hydroxybenzoic acid	y = 0.5959x + 0.4365		0.4 ± 0.0		1.3 ± 0.0			0.2 ± 0.0	1.2 ± 0.0
2	3.4	269	Phenolic acid	Gallic acid	y = 0.6442x − 0.4737			0.2 ± 0.0				0.2 ± 0.0	
3	7.7	222	Derivative of the p-hydroxybenzoic acid	3,4-Dihydroxy-5-methoxybenzoic acid	y = 0.1682x − 0.047	0.2 ± 0.1	21.1 ± 1.9	239.3 ± 20.2	142.2 ± 0.0			164.4 ± 3.6	98.8 ± 4.3
4	19.1	265	Derivative of methyl coumarin	L-Tyrosine 7-amido-4-methylcoumarine	y = 0.1483x − 0.2105		4.8 ± 0.6			0.2 ± 0.3			
5	19.9	266	Coumarin derivative	7-Hydroxycoumarin-3-carboxylic acid	y = 0.0799x + 0.1586					1.8 ± 0.9	6.3 ± 0.7		
6	22.6	230	Flavonoid	Rutin	y = 0.1029x + 0.6179	4.0 ± 1.8			2.2 ± 1.7			0.3 ± 0.0	
7	23.3	267	Stilbene	Polydatin	y = 0.0445x − 0.0083					0.5 ± 0.0			
8	35.2	340	Phenolic acid	2,4-Dihydroxy-3,6-dimethylbenzoic acid	y = 0.1612x − 0.1498								0.1 ± 0.0
9	42.1	286	Flavonoid	3′,5′-Dihydroxyflavone	y = 0.1267x − 0.0317		4.7 ± 0.0	3.6 ± 0.0					
10	43.4	281	Derivative of phenyl coumarin	5,7-Dihydroxy-4-phenylcoumarine	y = 0.1605x − 0.0115						0.5 ± 0.3		
11	44.1	240	Chalcone derivative	Cardamonin	y = 0.009x − 0.035						5.5 ± 3.9		
12	44.6	240	Stilbene	Pinostilbene	y = 0.041x + 0.0646					0.1 ± 0.0	1.0 ± 0.0		
13	44.9	259	Phenolic acid derivative	3-Benzyloxy-4,5-dihydroxy-benzoic acid methyl ester	y = 0.0961x + 0.5481	0.1 ± 0.0							
14	47.0	257	Stilbene	Pinosylvin monomethyl ether	y = 0.1265x − 0.5347	1.1 ± 0.5				0.7 ± 0.1			
15	47.9	262	Methoxyphenol	3, 6,3′-Trimethoxyflavone	y = 0.1017x + 0.1091					0.1 ± 0.0	0.5 ± 0.0	0.5 ± 0.0	

**Table 4:** Suggested chemical classes of some unidentified peaks detected in the leaf and fruit extracts of B. libanotica.

Compound Number	tR (min)	λmax (nm)	Suggested Chemical Class	Extract	References
Leaves					
L1	5	253	p-hydroxybenzoic acid	CHX	[11]
L2	7.5	240	Quercetin	CHX	[11]
L3	22.8	232	Flavonol	DCM	[11]
L4	44.3	233	Phenolic acid	CHX	[12]
Fruits					
F1	5	284	p-hydroxybenzoic acid	DCM	[11]
F2	7.5	255	Luteolin	CHX	[11]
F3	12.5	225	Flavanone	DCM	[11]
F4	30	336	Phenolic acid	CHX	[12]

**Table 5:** Identification of the volatile compounds of the *B. libanotica* extracts prepared from the leaves and fruits by GC-MS before and after derivatization.

			*B. libanotica*—Before Derivatization					
			Leaves			Fruits		
№	RI	Compound	CHX	DCM	MeOH	CHX	DCM	MeOH
1	-	permethyl 99A	+			+		
2	-	3-isopropylbenzaldehyde	+					
3	-	benzene, 2-methoxy-1,3,5-trimethyl-			+			
4	1850	neophytadiene	+	+		+	++	
5	1857	hexahydrofarnesyl acetone	+	+			+	
6	1893	3,7,11,15-tetramethyl-2-hexadecen-1-ol		+				
7	1930	methyl palmitate			+++			++
8	1937	7,9-di-tert-butyl-1-oxaspiro(4,5)deca-6,9-diene-2,8-dione	++	++			++	
9	1984	palmitic acid	+	++		++	++	
10	2127	phytol	+	+		+	+	
11	2132	methyl stearate			+			+
12	2157	linoleic acid	+	+			++	
13	2172	oleic acid		+		+++	+	
14	2372	4,8,12,16-tetramethylheptadecan-4-olide				+	+	
15	2422	phenol, 2,2′-methylenebis[6-(1,1-dimethylethyl)-4-methyl-	+	++	++		+++	
16	2520	2-monopalmitin	+++	++	+++		++	++
17	2736	α-monostearin		+				++
18	2878	α-tocospiro A	+	+		+	+	
19	2900	α-tocospiro B	+	+		+	+	
20	2902	octacosane	+++			+++	+++	
21	2914	glaucine		+			++	
22	2937	heptacosane	+					
23	2959	5-methylnonacosane	+					
24	2950	α-tocopherolquinone				+++		
25	2962	vitamin E	++			++		
26	3070	β-sitosterol		+		+++	+++	
27	3079	γ-sitosterol	+++					
28	-	didehydroglaucine		+			++	
29	-	β-amyrin				++	+	
30	-	stigmasta-3,5-diene	+					
31	-	lupeol	+			+++	+	
			***B. libanotica*—after derivatization**					
			**Leaves**			**Fruits**		
№	tR (min)	Compounds	CHX	DCM	MeCN	CHX	DCM	MeCN
1′	9.2	2,3-butanediol		+				
2′	9.8	carbamic acid				++		
3′	9.8	lactic acid		+++				
4′	10	hexanoic acid				++		
5′	10.3	glycolic acid		+++				
6′	10.4	1-butoxy-2-propanol	+					
7′	13.1	hydracrylic acid		+				
8′	21.6	octanoic acid				+		
9′	25.1	glycerol		++				
10′	27.2	thymol	+	+				
11′	29.2	butanedioic acid		+				
12′	29.4	carvacrol	+	+				
13′	31.3	glyceric acid		+				
14′	31.8	pyrrole-2-carboxylic acid				+		
15′	32.1	nonaoic acid	+					
16′	32.6	fumaric acid		+				
17′	33.2	cuminol	+					
18′	33.6	3-hydroxycaproic acid						+
19′	36.1	2-methyl-6-tertbutylphenol	+					
20′	36.2	malic acid		+				
21′	36.2	4-methylbenzoic acid	+					
22′	36.5	4-hydroxybutanoic acid		+				
23′	36.8	2,4-di-tert-butylphenol	+					
24′	37.2	(L) erythronic acid		+				
25′	37.3	tert-butylhydroquinone		+				
26′	37.5	(D) erythronic acid		+				
27′	37.7	(L) xylose		+				
28′	37.8	(D) xylose		+				
29′	37.9	3,4-dihydroxybenzaldehyde		++				
30′	38.1	4-hydroxybenzoic acid		+				
31′	38.3	D-xylonic acid		+				
32′	38.4	(D)-ribonolactone		+				
33′	38.44	dodecanoic acid	+	+				
34′	39.2	homovanillyl alcohol		+			+	
35′	39.3	levoglucosan					+	
36′	39.7	(L)-ribonolactone		++				
37′	39.9	vanillic acid	+					
38′	39.98	1-(3,4-dihydroxyphenyl) ethanone		+				
39′	40.1	loliolide	+					
40′	40.2	azealic acid	+	++				
41′	40.4	β-D-tagatopyranose						+
42′	40.5	2,5-furan diol		+				
43′	40.5	D-(-)-tagatofuranose						++
44′	40.6	D-psicofuranose « isomer 2 »			+			
45′	40.7	D-(-)-fructofuranose « isomer 2 »			+			
46′	40.76	myristic acid	+++	++				
47′	40.8	D-(-)-fructopyranose						++
48′	41.2	quininic acid		+				
49′	41.03	methyl α-D-glucofuranoside					++	
50′	41.4	syringic acid	+	++				
51′	41.5	α-D-mannopyranose			+			
52′	41.59	talose						+++
53′	41.7	pentadecanoic acid	+					
54′	42.1	D-gluconic acid		+++				
55′	42.3	L-gluconic acid		+++				
56′	42.4	β-D-(+)-mannopyranose			+			
57′	42.4	α-linolenic acid	+					
58′	42.5	palmitelaidic acid	+					
59′	42.4	β-D-glucopyranose						+++
60′	42.5	α,β-glucooctanoic γ-lactone		+				
61′	42.8	glucaric acid		+				
62′	43.2	ferulic acid					+	
63′	43.3	esculetin		+				
64′	43.6	heptadecanoic acid	+					
65′	43.7	caffeic acid		+++				
66′	43.76	1-octadecanol	+++					
67′	44.2	linoleic acid				+++	+++	
68′	44.5	stearic acid	+++	+				
69′	45.9	13-eicosenoic acid (Z)	+	+				
70′	46.1	arachidic acid	++	+				
71′	46.9	heneicosanoic acid	+					
72′	47	docosanol	+++					
73′	47.13	2-palmitoylglycerol	+					
74′	47.17	hernagine		+				
75′	47.4	1-monopalmitine	+++	+++				
76′	47.5	13-docosenoic acid (Z)	+	+				
77′	47.6	behenic acid	+++	+				
78′	47.7	N-methylhernagine		+++				
79′	47.8	(10E,15Z)-9,12,13-trihydroxy-10,15-octadecadienoic acid		+				
80′	48.1	sucrose						+
81′	48.37	2-monoolein				+		
82′	48.38	tricosanoic acid	+					
83′	48.4	tetracosanol	+					
84′	48.5	2-monostearin				+		
85′	48.6	monolinolein	+					
86′	48.66	1-linolenoylglycerol	+					
87′	48.67	2-monolinolenin					+	
88′	48.7	glycerol monostearate	++	+++				
89′	49	lignoceric acid	+++					
90′	49.6	2-phenylethyl β-D-glucopyranoside		+++				
91′	49.8	1-hexacosanol	+					
92′	50.2	γ-tocopherol	+					
93′	50.4	hexacosanoic acid	++					
94′	50.7	nonacosan-10-ol	+++					
95′	51.1	cajaninstilbene acid		+				
96′	51.13	methyl chlorogenate						+++
97′	51.14	1-octacosanol	++					
98′	51.3	chlorogenic acid		++				
99′	51.8	octacosanoate	++					
100′	52.03	hentriacontan-12-ol	+					
101′	52.2	campesterol	+					
102′	52.4	stigmasterol	+					
103′	53.5	α-amyrin				+		
104′	52.7	1-triacontanol	+					
105′	55.67	oleanolic acid	+					
106′	56.23	Ursolic acid	++					

+++: High presence; ++: average presence; +: low presence; RI is the retention index determined in regard to a series of n-alkanes (C_(7)_–C_(35)_) on the apolar DB-5 MS.

**Table 6:** Minimum inhibitory concentration (MIC) values obtained with the leaf extracts of *B. libanotica*.

Bacterial Strains	MIC (μg/mL)				
		CHX	DCM	EtOAc	MeOH
*Salmonella enterica* serovar Enteritidis	Gram (−)	19.5 ± 0	39 ± 1.2	-	-
*Salmonella enterica* serovar Kentucky		-	39 ± 0	-	-
*Salmonella enterica* serovar Infantis		-	19.5 ± 0	-	-
*Escherichia coli* ATCC 8739		9.7 ± 1.2	-	-	-
*Listeria monocytogenes* ATCC 19115	Gram (+)	19.5 ± 0	39 ± 0	-	-
*Listeria monocytogenes* Fish filet		-	-	-	-
*Staphylococcus aureus* ATCC 25923		-	-	-	-

**Table 7:** Minimum inhibitory concentration (MIC) values obtained with the fruit extracts of *B. libanotica*.

Bacterial Strains	MIC (μg/mL)				
		CHX	DCM	EtOAc	MeOH
*Salmonella enterica* serovar Enteritidis	Gram (−)	9.7 ± 1.2	39 ± 0	-	-
*Salmonella enterica* serovar Kentucky		2.4 ± 1.2	-	-	625 ± 0
*Salmonella enterica* serovar Infantis		-	-	156.2 ± 0	625 ± 0
*Escherichia coli* ATCC 8739		312.5 ± 1.2	625 ± 1.2	625 ± 0.4	625 ± 0
*Listeria monocytogenes* ATCC 19115	Gram (+)	2.4 ± 4.9	-	312.5 ± 0	625 ± 0
*Listeria monocytogenes* Fish filet		4.8 ± 1.2	-	-	-
*Staphylococcus aureus* ATCC 25923		-	-	78.1 ± 0	-

**Table 8:** Contributions of variable factors to the principal component analysis (%).

	F1	F2
TPC	22.6	1.0
DPPH	20.8	8.0
AChE	3.0	35.8
XOD	19.4	6.2
15-LOX	14.6	0.8
HCT-116	1.5	44.7
Caco-2	18.0	3.5

**Table 9:** Correlation established between variables and factors.

	F1	F2
TPC	0.9	−0.1
DPPH	0.8	−0.3
AChE	−0.3	−0.7
XOD	0.8	0.3
15-LOX	0.7	−0.1
HCT-116	−0.2	0.8
Caco-2	−0.8	−0.2

## Data Availability

Not applicable.
